# Monitoring the status of selected health related sustainable development goals: methods and projections to 2030

**DOI:** 10.1080/16549716.2020.1846903

**Published:** 2020-11-30

**Authors:** Kathleen Strong, Abdislan Noor, John Aponte, Anshu Banerjee, Richard Cibulskis, Theresa Diaz, Peter Ghys, Philippe Glaziou, Mark Hereward, Lucia Hug, Vladimira Kantorova, Mary Mahy, Ann-Beth Moller, Jennifer Requejo, Leanne Riley, Lale Say, Danzhen You

**Affiliations:** a Department of Maternal, Newborn, Child and Adolescent Health and Ageing, WHO, Geneva, Switzerland; b Global Malaria Programme, WHO, Geneva, Switzerland; c Department of Health Financing, WHO, Geneva, Switzerland; d Strategic Information Department, UNAIDS, Geneva, Switzerland; e Stop TB Department, WHO, New York, NY, USA; f Division of Data, Analytics, Planning and Monitoring, UNICEF, New York, NY, USA; g UN Department of Economic and Social Affairs, Population Division, New York, NY, USA; h UNDP/UNFPA/UNICEF/WHO/World Bank Special Programme of Research, Development and Research Training in Human Reproduction (HRP), Department of Sexual and Reproductive Health and Research, WHO, Geneva, Switzerland; i Noncommunicable Diseases Department, WHO, Geneva, Switzerland

**Keywords:** UN Sustainable Development Goals, maternal and under-5 mortality, incidence of TB, malaria, HIV, family planning, noncommunicable diseases

## Abstract

**Background**: Monitoring Sustainable Development Goal indicators (SDGs) and their targets plays an important role in understanding and advocating for improved health outcomes for all countries. We present the United Nations (UN) Inter-agency groups’ efforts to support countries to report on SDG health indicators, project progress towards 2030 targets and build country accountability for action.

**Objective**: We highlight common principles and practices of each Inter-agency group and the progress made towards SDG 3 targets using seven health indicators as examples. The indicators used provide examples of best practice for modelling estimates and projections using standard methods, transparent data collection and country consultations.

**Methods**: Practices common to the UN agencies include multi-UN agency participation, expert groups to advise on estimation methods, transparent publication of methods and data inputs, use of UN-derived population estimates, country consultations, and a common reporting platform to present results. Our seven examples illustrate how estimates, using mostly Bayesian models, make use of country data to track progress towards SDG targets for 2030.

**Results**: Progress has been made over the past decade. However, none of the seven indicators are on track to achieve their respective SDG targets by 2030. Accelerated efforts are needed, especially in low- and middle-income countries, to reduce the burden of maternal, child, communicable and noncommunicable disease mortality, and to provide access to modern methods of family planning to all women.

**Conclusion**: Our analysis shows the benefit of UN interagency monitoring which prioritizes transparent country data sources, UN population estimates and life tables, and rigorous but replicable modelling methods. Countries are supported to build capacity for data collection, analysis and reporting. Through these monitoring efforts we support countries to tackle even the most intransient health issues, including the pandemic caused by SARS-CoV-2 that is reversing the hard-earned gains of all countries.

## Background

The Millennium Development Goals (MDGs, 2000–2015) led to improved health and well-being through the collaborative efforts of United Nations (UN) agencies and their constituent Member States (MS). In 2015, the 2030 Agenda for Sustainable Development proposed 17 Sustainable Development Goals (SDGs) [1], one of which, SDG 3, *Ensure healthy lives and promote well-being for all at all ages*, calls for UN agencies with significant roles in improving health and development to be accountable for driving progress across a wide range of health areas and also strengthening health systems and financing. To promote measurement, the United Nations Statistical Commission (UNSC) created the Inter-agency and Expert Group on SDG Indicators (IAEG-SDGs). This group, composed of MSs and including regional and international agencies as observers, provides technical support for implementing the approved indicator and monitoring framework. It also regularly reviews methodological developments and issues related to the indicators and their metadata [2]. Some SDG 3 indicators were previously used to monitor the MDGs, while others are new, expanding the global development agenda into other health areas and supporting international development through measuring the strength of health systems (Table 1).

**Table 1.** Mapping of indicators for MDGs 4–8 with indicators for SDG 3, including lead custodian agencies and SDG tier designations (I–II)

**MDG 4** **Reduce child mortality**	**SDG 3 equivalent**	**SDG Custodian Agencies**	**SDG tier**
4.1 Under-five mortality rate	3.2.1 Under five mortality rate 3.2.2 Neonatal mortality rate	UNICEF	I
4.2 Infant mortality rate			
4.3 Proportion of 1 year-old children immunized against measles	3.b.1 Proportion of the target population covered by all vaccines included in their national programme	UNICEF/WHO	I
**MDG 5** **Improve maternal health**	**SDG 3 equivalent**	**SDG Custodian Agencies**	**SDG tier**
5.1 Maternal mortality ratio	3.1.1 Maternal mortality ratio	WHO	I
5.2 Proportion of births attended by skilled health personnel	3.1.2 Proportion of births attended by skilled health personnel	WHO and UNICEF	I
5.3 Contraceptive prevalence rate 5.6 Unmet need for family planning	3.7.1 Proportion of women of reproductive age (aged 15–49 years) who have their need for family planning satisfied with modern methods	United Nations Population Division of the Department of Economic and Social Affairs	I
5.4 Adolescent birth rate	3.7.2 Adolescent birth rate	United Nations Population Division of the Department of Economic and Social Affairs	I
5.5 Antenatal care coverage (at least one visit and at least four visits)			
**MDG 6** **Combat HIV/AIDS, malaria and other diseases**	**SDG 3 equivalent**	**SDG Custodian Agencies**	**SDG tier**
6.1 HIV prevalence among population aged 15–24 years	3.3.1 Number of new HIV infections per 1,000 uninfected population, by sex, age and key populations	Joint United Nations Programme on HIV/AIDS (UNAIDS)	I
6.2 Condom use at last high-risk sex			
6.3 Proportion of population aged 15–24 years with comprehensive correct knowledge of HIV/AIDS			
6.4 Ratio of school attendance of orphans to school attendance of non-orphans aged 10–14 years			
6.5 Proportion of population with advanced HIV infection with access to antiretroviral drugs			
6.6 Incidence and death rates associated with malaria	3.3.3 Malaria incidence per 1,000 population	WHO	I
6.7 Proportion of children under 5 sleeping under insecticide-treated bed nets			
6.8 Proportion of children under 5 with fever who are treated with appropriate anti-malarial drugs			
6.9 Incidence, prevalence and death rates associated with tuberculosis	3.3.2 Tuberculosis incidence per 1,000 population	WHO	I
6.10 Proportion of tuberculosis cases detected and cured under directly observed treatment short course			
	3.3.4 Hepatitis B incidence per 100,000 population	WHO	I
	3.3.5 Number of people requiring interventions against neglected tropical diseases	WHO	I
	3.4.1 Mortality rate attributed to cardiovascular disease, cancer, diabetes or chronic respiratory disease	WHO	I
	3.4.2 Suicide mortality rate	WHO	I
	3.5.1 Coverage of treatment interventions for substance use disorders	WHO, UNODC	II
	3.5.2 Alcohol per capita consumption (aged 15 years and older) within a calendar year in litres of pure alcohol (modified July 2020 UNSC)	WHO	I
	3.6.1 Death rate due to road traffic injuries	WHO	I
	3.9.3 Mortality rate attributed to unintentional poisoning	WHO	I
	3.A.1 Age-standardized prevalence of current tobacco use among persons aged 15 years and older	WHO, WHO FCTC	I
**MDG 7** **Ensure environmental sustainability**	**SDG 3 equivalent**	**SDG Custodian Agencies**	**SDG tier**
7.5 Proportion of population using an improved drinking water source 7.6 Proportion of the global population using an improved sanitation facility	3.9.2 Mortality rate attributed to unsafe water, unsafe sanitation and lack of hygiene (exposure to unsafe Water, Sanitation and Hygiene for All (WASH) services)	WHO & UNICEF	I
	3.9.1 Mortality rate attributed to household and ambient air pollution	WHO	I
**MDG 8** **Develop a global partnership for development**	**SDG 3 equivalent**	**SDG Custodian Agencies**	**SDG tier**
8.6 Sustainable access to affordable essential medicines	3.B.3 Proportion of health facilities that have a core set of relevant essential medicines available and affordable on a sustainable basis	WHO	II
	3.B.2 Total net official development assistance to medical research and basic health sectors	OECD	I
	3.8.1 Coverage of essential health services	WHO	I
	3.8.2 Proportion of population with large household expenditures on health as a share of total household expenditure or income	WHO	I
	3.C.1 Health worker density and distribution	WHO	I
	3.D.1 International Health Regulations (IHR) capacity and health emergency preparedness	WHO	I
	3.D.2 Percentage of bloodstream infections due to selected antimicrobial resistant organisms	WHO, pending final approval in March 2022	II

^1^As of the 51^st^ United Nations Statistical Commission, the global indicator framework does not contain any Tier III indicators; **Tier I**: Indicator is conceptually clear, has an internationally established methodology and standards are available, and data are regularly produced by countries for at least 50% of countries and of the population in every region where the indicator is relevant. **Tier II**: Indicator is conceptually clear, has an internationally established methodology and standards are available, but data are not regularly produced by countries. **Tier III**: No internationally established methodology or standards are yet available for the indicator, but methodology/standards are being (or will be) developed or tested.

^2^As of 17 July 2020; https://unstats.un.org/sdgs/files/Tier%20Classification%20of%20SDG%20Indicators_17%20July%202020_web.v2.pdf

The IAEG-SDGs classified all SDG indicators into three tiers based on their level of methodological development and the availability of data at the global level. The tiers are defined as follows: 1(I) indicators have internationally established methods and standards, and data are regularly collected by at least 50% of countries, 2(II) indicators are conceptually clear, with internationally established methodology and standards available, but data are not regularly produced by countries, and 3(III) indicators with no established methodology or standards, but work is ongoing to provide a sound methodology and standard. As of the fifty-first United Nations Statistical Commission, there are no Tier III indicators in the global indicator framework. The indicators chosen for this report are all Tier 1 (I) indicators (Table 1). They are but a fraction of the indicators (seven out of 27 indicators) designed to monitor SDG 3. Nonetheless, they provide the context for the UN Inter-agency joint work and reveal the partnership that has developed between the agencies to improve country data and estimation methods required to provide an unbiased examination of global progress towards reaching the SDG targets.

Custodian agencies – including the WHO, UNICEF, UNAIDS, UNFPA, UNDESA, OECD and the World Bank Group – are responsible for the global monitoring of the progress made on health-related indicators. The UN agency data sources for reporting estimates against SDG targets are mainly country collected data reported to the UN over the course of country-led monitoring activities. Although the UN agencies work together using the same data sources and estimates to monitor progress in a standard way, sometimes the public, governments, researchers and non-governmental organizations falsely believe that each agency uses different estimates. The confusion comes, in part, because agencies have different reporting and update cycles and may display the same data through different data visualizations on their respective websites [3]. To correct this misconception and show the results of our joint work together and with countries, we report on progress towards SDG targets for seven health indicators monitoring reproductive, maternal, newborn, child health, and communicable and noncommunicable disease burdens. These seven provide concrete examples of how UN Inter-agency groups work together with countries, academic and other institutions to provide transparent and publicly available data and estimates to monitor SDG 3. Projections to 2030 are available for these seven indicators, providing snapshots of the commitments needed from governments, donors, international organizations, non-governmental organizations and communities to meet the 2030 targets. We highlight common practices that facilitate validation of country-level data, support and engagement with countries to build better health information systems, and improve the health and well-being of their populations.

Although the current estimates show progress over time across all indicators, the projections all fall short of the 2030 SDG targets with many countries struggling to stay on track. The COVID-19 pandemic makes it unlikely that countries falling behind before the pandemic will be able to meet the targets by 2030 because of disruptions to essential health services for women, children, those living in malaria endemic countries and those living with HIV, TB, and noncommunicable diseases. COVID-19 will reverse recent progress and make achievement of 2030 targets more challenging.

## Methods

### Standard practices

All SDG health-related estimates benefit from multi-UN agency participation, independent expert groups to advise on methods, transparency of data sources and estimation and analysis methods, country consultations and a common reporting platform to present results and metadata. Projections of estimates based on annual average rates of change or best/worst-case scenarios are used to identify actions needed to meet 2030 goals.

Data for indicators on health outcomes come from three main country sources: population-based surveys, censuses and routine administrative systems (Civil Registration and Vital statistics, or CRVS). Countries differ in frequency, timeliness and completeness of data available from these sources for each indicator, making it difficult to compare results across countries, populations or time. Country collected data, adjusted to form population-level estimates, allow comparisons between populations of interest for monitoring purposes. Data adjustments use standard and reproducible methods compatible with the Guidelines for Accurate and Transparent Health Estimates Reporting (GATHER) [4], which defines best practices for calculating health estimates for multiple populations using multiple information sources [4]. The GATHER checklist strives to ensure that each estimation process is reproducible by other interested parties. The Inter-agency groups responsible for the estimates publish in both scientific journals and in Inter-agency or intergovernmental reports adhere to GATHER guidelines.

All UN Agency estimation processes presented in this paper follow similar guiding principles, including GATHER, but also align on: (1) the use of United Nations Population Division of the Department of Economic and Social Affairs (UN DESA) population estimates and projections for denominators and numbers of live births and use of life tables jointly constructed by WHO and UN Population Division [5,6]; (2) the importance of official country consultations on estimates derived from nationally representative data or data predicted from co-variates associated with the outcome of interest; and (3) a shared commitment to improving country capacity to report health data in an accurate and timely manner so that over time the data reported by the Inter-agency groups moves closer to country reported data and farther from estimates based on model algorithms.

No UN Agency publishes country-level health estimates without a formal Member State consultation [7]. Member States nominate focal points to review the estimates. Normally these focal points are from a country’s national statistical agency, but some also have a representative from the Ministry of Health. There are also SDG focal points from the UN statistical Division’s IAEG Secretariat that can review country estimates in the absence of a country-nominated focal point. The country consultation process gives each country’s ministry of health, national statistics office or relevant agency the opportunity to review all data inputs, the estimation methodology and the draft estimates. These reviews have resulted in additional or updated data being added to the models to improve the final estimates, and also provide an opportunity to engage countries and build capacity for estimation. Equally important, they provide an opportunity to discuss the gaps and quality of existing data, and encourage countries to improve the availability and quality of their own data.

### Methods for estimations and projections

The Inter-agency groups use statistical models to develop (or in the case of HIV, support countries to develop) estimates to monitor health-related outcomes. The process for each modelling exercise is similar; each group compiles relevant, available nationally representative data, assesses these data for quality, and uses them in a statistical model to develop the estimates. If the assessment of data inputs and quality deems it necessary, adjustments are made by applying standard methods. For some indicators, statistical models fitted to the data generate a smooth trend curve that averages potentially different estimates coming from different data sources for a country. For others, common covariates, added to the model, develop estimates for countries with little or no data. The statistical model is then used to extrapolate values to a target year. These processes provide continuity of the estimates over time while allowing for advances in methodology to be incorporated into the estimates in a systematic manner.

Table 2 displays a summary of each of the seven indicators, their targets for 2030, interagency technical group, types of models used for the estimates and projections and links to published method descriptions. In addition, each SDG goal has target(s) and indicator(s) with defined metadata and methodology for analysis and/or development of comparable health estimates. These data, methods, and their limitations are available to the public on the UN Statistical Division’s database found at https://unstats.un.org/sdgs/indicators/database/.

**Table 2.** Methodological description for 7 SDG 3 indicators showing the interagency or technical group responsible for developing the estimation and projection models and a link to the methods for each indicator

SDG indicator	Indicator	Target for 2030	Interagency/Technical group	Model used for estimate	Model used for projections	Link to model and methods
3.1.1.	Maternal mortality ratio (number of maternal deaths per 100,000 live births).	Reduce the global maternal mortality ratio to less than 70 per 100,000 live births.	United Nations Maternal Mortality Estimation Inter-Agency Group (MMEIG), (WHO (lead), UNICEF, UNFPA, the World Bank Group and the United Nations Population Division of the Department of Economic and Social Affairs	**Bayesian models (BMat)** 1. The CRVS model (for countries that have functioning CRVS systems)- a Bayesian CRVS adjustment model is used to account for errors in reporting of maternal deaths and to obtain adjustment factors. 2. The BMat model (for all countries)- a Bayesian maternal mortality estimation model is used to estimate the MMR for each country year of interest.	Scenario-based projection using the median annual rate of reduction (1.7%) from the recent estimation round (2000–2017).	https://www.who.int/reproductivehealth/publications/maternal-mortality-2000-2017/en/.
3.2.1	Under-5 mortality rate per 1,000 live births	End preventable deaths of newborns and children under 5 years of age, with all countries aiming to reduce neonatal mortality to at least as low as 12 per 1,000 live births and under-5 mortality to at least as low as 25 per 1,000 live births.	UN Inter-agency Group for Child Mortality Estimation (UN-IGME) (UNICEF (lead), WHO, World Bank Group, the United Nations Population Division of the Department of Economic and Social Affairs)	Bayesian B-splines bias-adjusted model (B3 model)	Scenario-based projection using average annual rate of change in U5MR from 2000 to 2019 in each country	www.childmortality.org
3.3.1	Number of new HIV infections per 1,000 uninfected population, by sex, age and key populations	End the epidemics of AIDS, tuberculosis, malaria and neglected tropical diseases and combat hepatitis, water-borne diseases and other communicable diseases.	Reference Group on Estimates, Modelling and Projections (UNAIDS (lead), UNICEF, WHO, US Government PEPFAR programme and the Global Fund to fight AIDS, TB and malaria)	Country teams use UNAIDS supported software, Spectrum	Goals model in Spectrum	https://www.unaids.org/en/dataanalysis/knowyourresponse/HIVdata_estimates; www.epidem.org;
3.3.2	Tuberculosis incidence per 100,000 population	End the epidemics of AIDS, tuberculosis, malaria and neglected tropical diseases and combat hepatitis, water-borne diseases and other communicable diseases.	WHO Task Force on TB Impact Measurement (WHO (lead), Experts in TB epidemiology, statistics and modelling, representatives from major technical and financial partners)	Prevalence surveys; Notifications from high-income countries adjusted for under-reporting and under-diagnosis; Case notifications combined with expert opinion about case detection gaps	TB-MAC modelling consortium	http://tb-mac.org/
3.3.3	Malaria incidence per 1,000 population	End the epidemics of AIDS, tuberculosis, malaria and neglected tropical diseases and combat hepatitis, water-borne diseases and other communicable diseases	WHO’s Malaria Policy Advisory Committee (WHO, (lead), WHO’s Global malaria programme with inputs from endemic countries, donor agencies, and academic institutions)	spatio-temporal Bayesian geostatistical model that includes programme, environmental, and sociodemographic covariates.	Projection based on current trend	who.int/malaria/publications/world-malaria-report-2019/en/
3.7.1	Proportion of women of reproductive age (aged 15–49 years) who have their need for family planning satisfied with modern methods	Ensure universal access to sexual and reproductive health-care services, including for family planning, information and education, and the integration of reproductive health into national strategies and programmes	Expert group working to produce the estimates of family planning indicators (the United Nations Population Division of the Department of Economic and Social Affairs (lead), external experts and statisticians)	Bayesian hierarchical model combined with country-specific time trends	Bayesian hierarchical model extrapolation of the systematic logistic trends and the autocorrelated error processes	https://www.un.org/development/desa/pd/data/estimates-and-projections-family-planning-indicators.
3.4.1	Mortality rate attributed to cardiovascular disease, cancer, diabetes or chronic respiratory disease.	Reduce by one third premature mortality from noncommunicable diseases through prevention and treatment and promote mental health and well-being	NCD Countdown 2030 (WHO (lead), The Lancet, NCD Alliance, the WHO Collaborating Centre on NCD Surveillance and Epidemiology at Imperial College London, and researchers and practitioners from all regions)	Unconditional probability of dying from 4 main NCDs using a life table method calculating the risk of death between 30 and 70 years and cause of death estimates from WHO Global Health Estimates	A linear trend line is fitted to the unconditional probabilities of death from 2010 to 2016 and its slope is used to provide the value for the average annual rate of change	www.ncdcountdown.org

**Figure 1.**
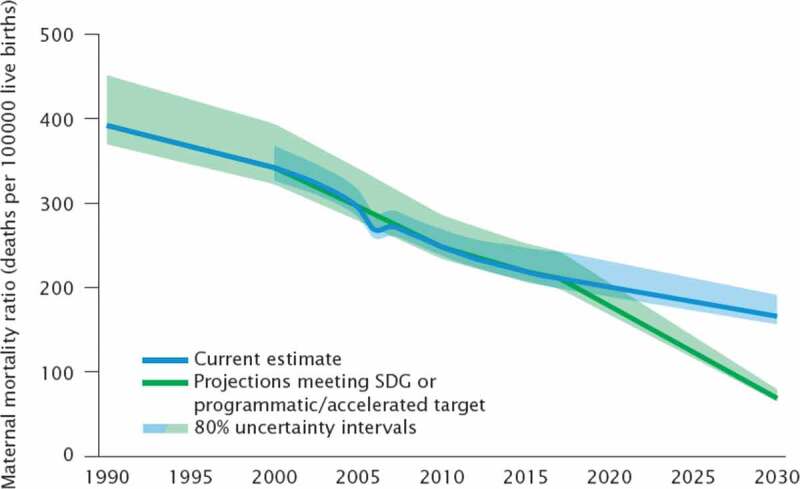
Comparison of actual and projected progress in maternal mortality ratio (MMR) over time

The results section shows the scenario-based projections to 2030 and demonstrate how progress towards the targets is being monitored. Figure 1 through 7 describe the estimated progress that has been made globally across the indicators. Blue lines represent the current estimates to the latest year with a shaded area of the same color representing the uncertainty intervals for the estimates. Projections based on past trends are presented as black lines and projections of meeting the SDG targets are shown in green.

### Maternal mortality ratio

The maternal mortality ratio (MMR) is the number of maternal deaths during a time-period per 100 000 live births during the same time period. The risk of a maternal death is quantified relative to the number of live births (1). Bayesian models are used for the estimation of MMR (Table 2)[8].

The global target for MMR is less than 70 maternal deaths per 100,000 live births by 2030. A scenario-based projection made for 2030 highlights the impact on maternal survival of meeting SDG targets by 2030 [9]. Country-specific annual rates of reduction (ARR) from 2000 to 2017 were used to reflect recent annual rate of reduction (ARR) and represent what would happen if the typical country ARR continues until 2030. The ARR selected is 1.7%, and represents the median ARR from the recent estimation round.

### Under-5 mortality rate

Estimates of neonatal, infant, under-5 and 5 to 14-year-old mortality are updated annually after a review of new data and an assessment of data quality by the UN Inger-agency group for child mortality estimation. The estimates are based on available nationally representative data from censuses, surveys and vital registration systems. Covariates are not used to derive estimates, but a statistical method, accounting for data biases, is applied to empirical data to generate trend estimates after a data quality assessment. Estimation and projection of under-5 mortality rates use the Bayesian B-splines bias-adjusted model (B3 model), already developed, validated, and used to produce previous rounds of the UN IGME child mortality estimates (Table 2)[10–12].

Projections to 2030 are made for the following scenarios: (1) current trends continue scenario: based on the observed average annual rate of change in U5MR in 2000–2019 in each country; (2) SDG targets met scenario: projections use the required average annual rate of reduction to achieve the SDG target given the current U5MR in 2019 in a country. If a country has already met the SDG target in 2019 or will meet the SDG target based on scenario 1, projections assume that current trends continue. Projections are done at country level and then aggregated to regional and global levels. Uncertainty intervals (90%) in the projections were calculated by projecting each 8,000 sample trajectory from the posterior distribution forward and taking the 5th and 95th percentile for each year of the projection period.

### HIV/AIDS

The UNAIDS Reference Group on Estimates, Modelling and Projections publishes global estimates of number of new HIV infections per 1,000 uninfected population, by sex, age and key populations [13]. Country teams provide national estimates using UNAIDS supported software. The Fast Track modelling Group, developed calculations for the projection scenarios shown in Figure 3. The methodology for the estimates can be found at www.epidem.org.

### Tuberculosis

Using the advice of the WHO Task Force on TB Impact Measurement, WHO produces annual estimates of the burden of disease caused by TB, measured in terms of incidence, prevalence and mortality. Sources of data include surveillance systems (case notifications and death registrations), special studies (including surveys of the prevalence of disease), mortality surveys, ‘inventory studies’ of under-reporting of detected TB, in-depth analysis of surveillance and other data, expert opinion and consultations with countries.

Projections were adopted as part of the End TB Strategy endorsed by the World Health Assembly in 2014. The overall goal of the End TB Strategy is to ‘End the global TB epidemic’. It sets ambitious targets for reductions in TB deaths, and cases for 2030. The projections were developed in collaboration with the TB-MAC modelling consortium (Table 2), and through wide consultations conducted in 2013–2014 [14].

### Malaria

Estimates of malaria incidence are made using inputs from endemic countries, donor and academic institutions. Endemic countries have annual estimates, made within one year of a calendar year’s end, for example, estimates made in 2020 will be for year 2019 using data reported from the country for 2019 [15,16]. Estimates are shared with malaria programme personnel with feedback included before publication. One of two methods are used to estimate the number of malaria cases, depending on transmission status and location of the country (Table 2).

### Family planning

Estimates and projections of family planning indicators, including the proportion of women of reproductive age (aged 15–49 years) who have their need for family planning satisfied with modern methods, are updated annually after a review of new data and an assessment of data quality. The expert group, including external academic experts and statisticians, and demographers and statisticians from the United Nations Population Division, developed the Bayesian hierarchical model used to produce the estimates [17] (Table 2). The projections are obtained from the fitted Bayesian model by extrapolation of the systematic logistic trends and the autocorrelated error processes.

### Noncommunicable diseases (NCDs)

The risk of premature death from the four main NCDs is estimated using the unconditional probability of dying from any of these NCDs. Unconditional probability is the probability of death in the absence of competing causes of death, and uses life table methods for age-specific death rates from the causes of interest. Age-specific death rates for the combined four cause categories (typically in terms of 5-year age groups 30–34, …, 65–69). A life table method allows calculation of the risk of death between exact ages 30 and 70 from any of these causes, in the absence of other causes of death. The ICD codes included in the calculation are cardiovascular disease: I00-I99; cancer: C00-C97;‬‬‬‬ diabetes: E10-E14; and chronic respiratory: J30-J98. Cause of death data comes from the WHO Global Health Estimates (GHE) [18].

Average annual rate of change was used to produce the projections in Figure 7. A linear trend line is fitted to the unconditional probabilities of death for years 2010–2016, and the slope of this line is used to provide the value for average annual rate of change. The linear trend is used to account for the distribution of reductions in absolute NCD mortality over time, because this depends on the interventions introduced by countries, and their associated lag times between intervention and mortality reduction (Table 2)[19].

## Results

The results of the scenario-based projections are presented below for each indicator.

### Projections for maternal mortality ratio

The maternal mortality ratio (MMR) estimates are developed for each country, WHO regions, SDG regions, UNICEF and UNFPA regions, World Bank Income Groups as well as global figures. Globally, an estimated 290, 000 (uncertainty interval (UI) 80%:279,000 to 340,000) maternal deaths occurred in 2017, providing an overall MMR of 211 (UI 80%: 199 to 243) maternal deaths per 100,000 live births.

Figure 1 shows the results of the estimates for 2017, current trend line (in blue) and the projections to 2030 with the target trend line (in green). The green line represents the reduction necessary for each country to meet the global SDG target of less than 70 maternal deaths per 100,000 live births by 2030. The shaded area of the graphs represent 80% confidence intervals reflecting the nature of maternal mortality as a rare event, with its estimation subject to substantial uncertainty [8].

The projections show that if the world reduced the global MMR to less than 70 deaths per 100,000 live births by 2030, there would be 89,000 maternal deaths in 2030 and almost 2.5 million cumulatively between 2016 and 2030 (Figure 1). This projection is much lower than the current projected trend in MMR which uses the observed global AAR of 1.7% and leads to an MMR of 142 per 100,000 live births.

### Projections for U5MR

The SDG target for U5MR is country specific. 122 countries have already met the SDG target of at least as low as 25 deaths per 1,000 live births and 20 countries will do so by 2030 if current trends continue based on the point estimate without considering uncertainty intervals. A remaining 53 countries, almost three quarters of which are in sub-Saharan Africa, need accelerated progress to meet the target by 2030. Because many countries already have an U5MR lower than the SDG target, the global U5MR target will be lower than 25. Currently, the suggested global target is 16.6 deaths per 1,000 live births.

**Figure 2.**
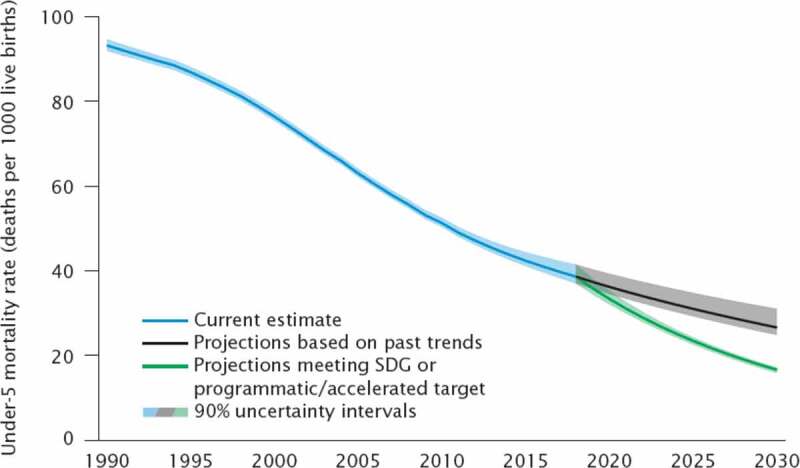
Comparison of actual and projected progress in under-5 mortality rates over time

Figure 2 shows the current estimates from 1990 to 2019 (blue line) with scenario 1, the projections based on current trends (black line) and scenario 2, the result of meeting the SDG target by 2030 (green line). Comparing the results of the two scenarios, it is obvious that many countries need to accelerate progress in reducing the under 5 mortality rate and that the world will not meet the global SDG target if current trends continue (Figure 2). More than 50 countries need to accelerate reductions in under-5 mortality to reach the SDG target, almost three quarters of these are in sub-Saharan Africa where under-5 mortality rates are highest at 78 deaths per 1,000 live births [12].

Accelerated progress to meet the SDG target in the countries that are falling behind would mean averting almost 11 million under-5 deaths compared with the current trends continue scenario. If current trends continue, around 48 million children under 5 years of age will die between 2020 and 2030, half of these will be newborns.

### Projections for HIV

**Figure 3.**
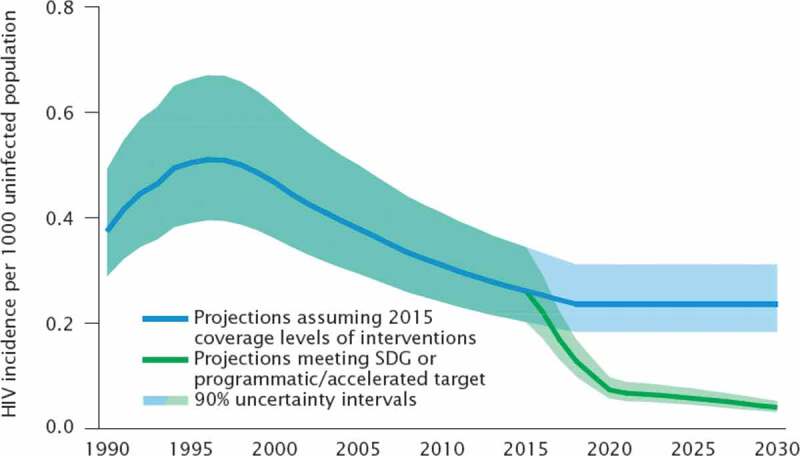
Comparison of actual and projected progress in new cases of HIV/AIDS per 1,000 uninfected population over time

Figure 3 shows estimates from 1990 to 2019 (blue line), with projections assuming 2015 coverage level for HIV prevention interventions (continuing blue line) and projections towards meeting the SDG target or accelerating progress (green line). The optimistic scenario (green line) is based on scaling up prevention and treatment interventions in individual countries using the Goals model, a module within the Spectrum model which is used for the estimates. The projections show that based on current estimates of new cases to 2030, the world will fall short of ending the HIV epidemic by 2030 (Figure 3). Only by meeting programmatic targets will countries end HIV as a public health threat by 2030.

**Figure 4.**
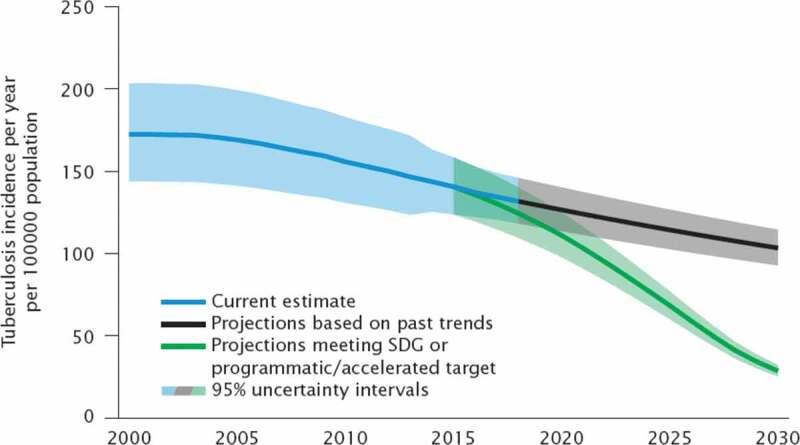
Comparison of actual and projected progress in tuberculosis incidence per year per 100, 000 population over time

Programme targets for 2025 and corresponding updated projections of epidemiological impact are being developed in 2020.

Every two years UNAIDS trains approximately 500 national epidemiologists, monitoring and evaluation officers, development partners, and others to use the Spectrum model. A six person team at UN AIDS manages the estimate process, covering all aspects from methodological developments to tracking correspondence with countries. Other partners support the process by providing facilitators to country workshops, contributing data and ideas to the Reference Group and also contributing to the costs of the workshops and capacity building in countries.

### Projections for tuberculosis incidence

Figure 4 shows that based on current trends for 2015–2018, the decline in TB incidence is insufficient to meet the SDG target 3.3 by 2030. The first scenario (black line), shows the projected tuberculosis incidence if the current trends continue to 2030. The projected scenario (green line), represents an 80% reduction of incidence rate by 2030 compared with 2015, in line with the SDG target 3.3 to eliminate TB by 2030.

Strengthening national TB surveillance and CRVS (Civil Registration and Vital Statistics), and the data they produce, is the only credible way to ensure robust planning and effective monitoring of progress towards global and national targets for TB. Settings with universal health coverage and TB surveillance systems where most new TB cases are detected and reported, constitute the benchmark to which National TB Programmes (NTP) can be compared. WHO is coordinating the global effort to identify and address performance gaps using a WHO TB surveillance checklist of standards and benchmarks assessment for national notification and vital registration systems [20].

### Projections for malaria incidence

**Figure 5.**
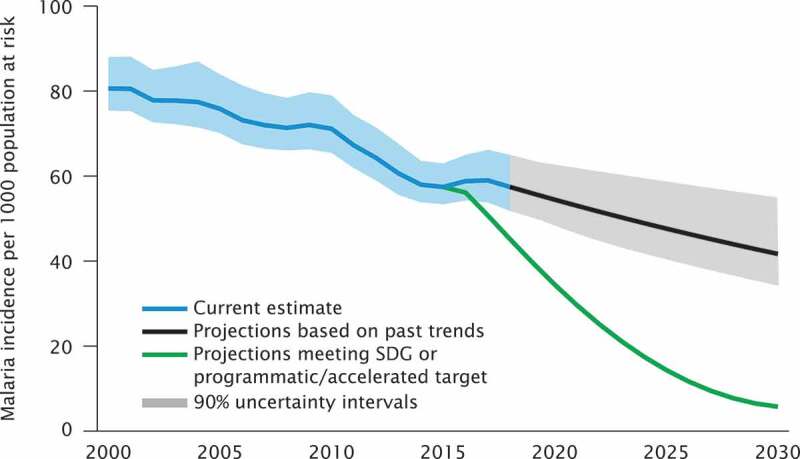
Comparison of actual and projected progress in malaria case incidence over time

Figure 5 shows scenario-based projection; (1) projections based on past trends (black line), and (2) projections meeting SDG or accelerated target (green line).

Globally, 30 countries in sub-Saharan Africa account for about 90% of the burden of malaria morbidity and mortality. The global incidence rate, measured as number of cases per 1,000 population fell from 71 in 2010 to 57 in 2018 [15], since then it has stalled. While the gains to-date are impressive, globally, the 2020 GTS milestones [16] for morbidity will not be achieved. To meet the 2020 GTS milestones, 2018 needed to have a global malaria case incidence of 45 per 1000 population at risk instead of the current estimated incidence of 58 cases per 1000 population at risk. Accelerated change is also needed on the current trajectory to meet the 2025 and 2030 milestones. On current trends in incidence, estimated malaria incidence will be 55 in 2020, 48 in 2025 and 43 in 2030, instead of the 35, 14 and 6 required for achievement of GTS milestones. If global investment in malaria is interrupted, and without a significant increase in domestic resources, global trends may revert to the peak transmission years before accelerated funding for malaria. Figure 5 shows a comparison of current trends with GTS targets.

**Figure 6.**
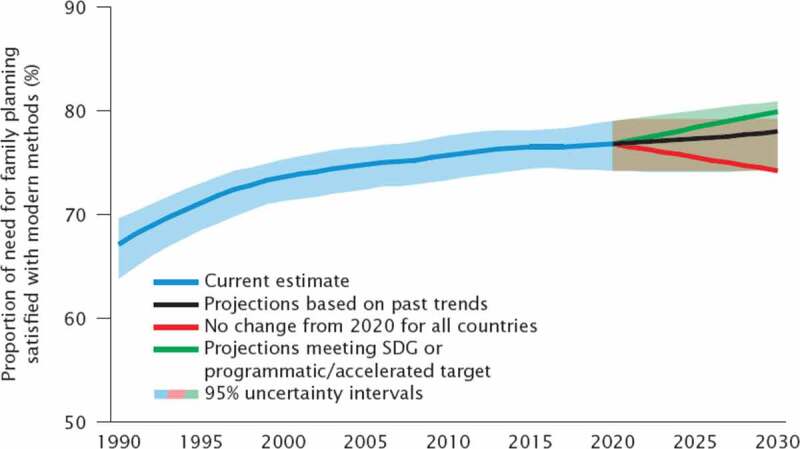
Comparison of actual and projected progress in proportion of woman of reproductive age (15–49 years old) who have their need, or demand, for family planning satisfied by using modern methods of contraception

### Projections for the need for family planning satisfied with modern methods

Globally, the proportion of women of reproductive age (15 to 49 years old) who have their need for family planning satisfied with modern contraceptive methods has increased slightly, changing from 74% in 2000 to 76% in 2019 [21]. In 43 countries, of which 32 are low-income countries, less than half of the need for family planning is met by modern methods.

Figure 6 shows projections based on past trends (black line); projections assuming no change on 2020 estimates (red line) and projections based on accelerated progress (green line). The projections show that if current trends continue, by 2030 only 77% of women aged 15 to 49 years will have their need for family planning satisfied by using modern methods of contraception. An accelerated progress based on the 90th percentile of the projected values of the indicator for the world in 2030 is around 80%. Under the scenario of no changes since 2020 in all countries, the proportion of the need for family planning satisfied by modern methods would decline globally to 74% in 2030, because in the coming decade, many countries with largest gaps in meeting family planning needs are expected to experience rapid growth in the numbers of women of reproductive age with need for family planning due to a combination of high population growth and changing childbearing intentions.

### Projections for NCDs

The probability of dying prematurely from an NCD (cancer, cardiovascular disease or chronic obstructive pulmonary disease) has decreased since 2000, largely due to increased international attention to the leading risk factors for these diseases, tobacco consumption, overweight/obesity, raised blood pressure and lipid levels and high alcohol consumption.

**Figure 7.**
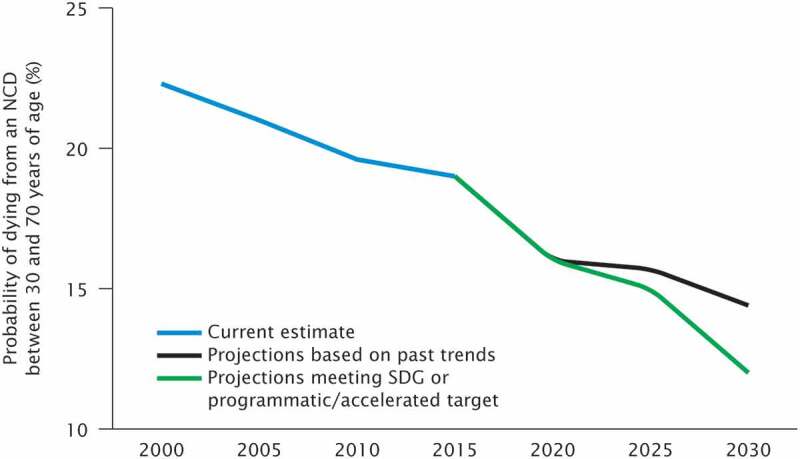
Comparison of actual and projected progress in global probability of death from NCDs between ages 30 to 70 years

Figure 7 shows the current estimate (blue line), projections based on past trends (black line), and projections based on average annual rate of change needed to meet the SDG target (green line). The projections show that the trend based on observed estimates of probability of dying from an NCD between the ages of 30 and 70 years falls short of the SDG target for a reduction of one-third of premature mortality from NCDs by 2030.

## Discussion

We describe how UN agencies work together to ensure coherent use of data sources and similar estimation methods to produce estimates and projections for selected SDG indicators, including consultation with experts and countries, and transparency of data sources and analysis methods to ensure high quality of monitoring of the SDG targets. The value of this work lies in the commitment to standard modelling methodology using national data sources that are provided transparently so that others may replicate the work. The commitment to transparency and maintaining a standard protocol stems from the over-arching vision of building country capacity to collect, analyse and disseminate health data to drive action and accountability in countries. High-quality results using standard and replicable methods build confidence in the estimates and provide incentives for government action. The projections allow national governments and other stakeholders to see where more action, advocacy, and support is needed to meet targets and to prevent premature mortality and improve health outcomes. Our analyses show that progress has been made over the past decade in the seven example health-related areas. Nonetheless, the targets set for 2030 are challenging. Based on current trends none of the associated SDG targets of the seven selected indicators will be achieved.

The key benefits in having UN agencies at the center of the monitoring is to galvanize country action. Country consultations, a cornerstone to the interagency estimation processes, ensure that estimates are derived using all available country data meeting inclusion criteria. Countries are active participants in the process by reviewing and revising their estimates, providing new data and ultimately decreasing the need to have model-based estimates. More processes in which countries develop their own estimates, similar to those used for the HIV estimates, should be encouraged to promote country data ownership and action. However, the HIV field had considerable resources available during the 2000s and as a result it was possible to train a cadre of country epidemiologist on the models that are used to create the HIV estimates. These trainings cost approximately US$600,000 every two years (covering the costs to bring one or two national epidemiologists to a regional workshop in 11 different regions). It is unlikely that other inter-agency groups will be able to systematically afford this type of intensive training despite the best efforts of all UN partners.

There are other global health estimation efforts reporting on progress towards the same SDG targets [22]. These are led by academic institutions and consortiums often funded from the same sources. They compete with UN led efforts yet are strongly supported by global donors and medical journals, including the Bill and Melinda Gates Foundation (BMGF) and the Lancet, who leverage the competing estimates to focus attention on global public health and health statistics-related issues and their own products [23]. As much as the attention to global public health is welcomed, the continued investments and focus on new and improved estimation processes and projection methodologies does little to build country capacity to systematically collect and report on actual health data and prolongs the use of estimates in the place of functional health information systems for all countries.

Regardless of which organization or experts produce the metrics, the same, sometimes limited, data sources are used to produce estimates although modelling methods vary. The UN agencies curate these data sources and provide them to all researchers as a global public good. The results from other estimation efforts may or may not approximate the UN interagency results depending upon the demographic parameters, assumptions and models used. A balance is needed between the focus on novel methodologies requiring time intensive, expensive computing and standard, replicable methods that use country data and can be done by national epidemiologists [24]. Examples of harmonized modelling methods employed by the UN inter-agencies include use of standard life tables and population estimates from the UN Population Division’s World Population Prospects (WPP) and the use of websites such as www.childmortality.org to provide details of country data sources and models used to monitor SDG 3.2.1 and 3.2.2, under-5 mortality and neonatal mortality respectively. It is the mandate of the UN system to produce consistent and replicable numbers for global monitoring of SDGs that countries can use to drive progress. Efforts are needed to improve the transparency of other modelling work so that any differences in results across them can be clearly conveyed and also to avoid distracting arguments about which numbers best reflect current status and progress for countries, regions and the world.

Recently there has been greater interest in strengthening routine health information systems not only to estimate coverage of care but also mortality [25]. As these systems mature, it is critical that methods used to derive estimates from them include data quality assessment steps and standardized approaches. In 2019, WHO launched an initiative with eleven other agencies, the Global Action Plan (GAP) for health-related SDGs, aiming to strengthen support to countries to achieve the health-related SDG targets [26]. WHO will take the lead and engage other UN partners in the area of data and digital health, acknowledging that improving data systems for health is a key component that needs to be strengthened [26]. This is especially true for the countries that lack recent data for the SDG indicators. Evidence suggests that fewer than half of all countries have data for 40% of the health-related SDG indicators, signaling a need to strengthen country capacity building for data collection, management and analysis [27].

The Health Data Collaborative (HDC) was launched in 2016 to strengthen country health information systems, and help meet the challenge of monitoring health and health-related SDGs. Partners include UN agencies (UNICEF, WHO, the World Bank Group, and UNAIDS), academic institutions from around the world, global public health funders and country partners working together to enhance country capacity, improve efficiency and alignment of investments in health data systems and increasing the impact of global public goods on health data systems through sharing, learning and country engagement. Incorporating methodological exercises, such as the ones presented here, in regional and national workshops using country-level results will help build capacity of local researchers to monitor country progress towards achieving the SDG targets nationally. The HDC could take a leadership role in promoting information exchange between the UN Interagency groups and Member States by holding workshops to extend the use of best practices used by some technical groups to other indicators. Examples include the UNAIDS-supported country teams that produce national estimates of HIV incidence using UNAIDS supported software and the UN-IGME website for presenting the data sets and model used to produce national under-5 mortality estimates and making these accessible to all stakeholders, www.childmortality.org.

The SARS-Cov-2 pandemic is challenging health systems everywhere with many countries unable to meet the demands for patient care for COVID-19 and for essential services. At the same time, health services for reproductive, maternal and child health and identification and treatment of HIV/AIDs, tuberculosis, malaria, and NCDs are on-going daily events that are part of the SDG monitoring to improve population health outcomes described in this paper. If COVID-19 disease results in wide spread disruption to essential health services, increases in mortality from causes other than COVID-19 disease can be expected [28]. Countries burdened by the pandemic may experience reversals in progress and be unable to meet SDG health-related targets. The Global Fund’s COVID-19 Response Mechanism has allocated US$1 billion to help mitigate impacts on TB, HIV and malaria, an amount that may not fully cover future needs.

It is even more critical for UN agencies to work together with Member States to monitor the effects of the pandemic on SDG progress and identify where additional support and resources are needed. Many modelling efforts are on-going to determine just what the effects of the pandemic may be on health outcomes. It is especially important that all groups estimating the effect of COVD-19 disease on health outcomes make their methods and data sources transparent so as not to undermine public trust in data about the pandemic [29]. The process of monitoring SDG targets and country progress is not just an academic exercise to improve methods for modelling of health data, but instead an important way to build country accountability for improving health information systems. The results presented here highlight the importance of working together across UN agencies at the global level by harnessing academic expertise while also fostering country capacity.

Now more than ever, instead of investments in academic exercises to improve methods for modelling of health data, we need more investments in country health monitoring systems and greater country ownership of the measurement and accountability agenda to ensure context-specific actions to improve health and measurement. This is crucial because the choices that governments make now will affect the long term health outcomes of individuals and populations [30] and also their ability to strengthen their national health systems based on actual data. These actions are urgent to ensure that the current pandemic does not stop the pre-pandemic progress made reported on here, even if it is insufficient to meet the targets for 2030.

## Paper context

We highlight UN Interagency efforts to estimate and project the results of 7 health-related SDG indicators. UN monitoring promotes transparency of data sources and methods, supporting harmonized tracking and improvement of national data collections. The goal is to empower countries to use national data to formulate health policies and make progress towards SDG 2030 targets in spite of COVID-19. All indicators show good progress but the 2030 targets will not be met without accelerated investment.
